# Engineering CAR‐Tregs with Phage‐Selected scFv Enables a New Paradigm for Immune Regulation

**DOI:** 10.1002/bies.70164

**Published:** 2026-07-15

**Authors:** Fatih Noyan, Matthias Hardtke‐Wolenski

**Affiliations:** ^1^ Dept. of Gastroenterology Hepatology, Infectious Disease and Endocrinology Hannover Medical School Hannover Germany; ^2^ Inst. of Medical Microbiology University Hospital Essen University Duisburg‐Essen Essen Germany

**Keywords:** cell biology, chimeric antigen receptor, FOXP3, immune system, immune tolerance, monoclonal antibody, scfv antibodies, Treg cell

## Abstract

Regulatory T cells (Tregs) are central to immune tolerance, yet antigen‐specific cell therapies lag behind cytotoxic CAR‐T approaches. Most CAR‐Treg programs still use single‐chain variable fragments (scFvs) derived from monoclonal antibodies optimized for effector function, a bias that may promote CAR clustering, tonic signaling, and lineage instability. Here, we propose a phage‐first binder discovery framework for CAR‐Tregs and state testable hypotheses linking phage‐display selection pressures to scFv biophysical properties and Treg fate. We hypothesize that multi‐parameter selection and counter‐selection can enrich scFvs with moderate affinity, low polyspecificity, and improved framework stability, thereby lowering tonic signaling in defined backbone architectures and preserving FOXP3/TSDR stability under inflammatory stress. We outline a falsifiable two‐phase roadmap—bench triage followed by mechanistic and preclinical validation to determine how scFv properties, expression level, and intracellular signaling domains define an optimal tonic window for durable immune regulation.

## Graphical abstract

Phage‐first CAR‐Treg design links binder discovery, scFv selection, and functional testing. Iterative selection and feedback may identify antigen‐binding domains with low tonic signaling, preserved FOXP3/TSDR stability, and effective antigen‐dependent suppression, supporting rational development of tolerance‐oriented CAR‐Treg therapies.

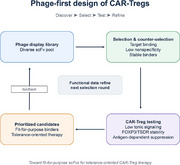

## 1 Introduction

Chimeric antigen receptor (CAR) engineering has revolutionized treatment of hematologic malignancies and is now expanding into non‐oncology indications. A parallel and increasingly compelling line of work seeks to harness regulatory T cells (Tregs) for durable immune tolerance in transplantation and autoimmunity. Against this backdrop, CAR‐modified Tregs (CAR‐Tregs) have emerged as a rational convergence: they offer antigen‐specific control over inflammation while preserving systemic immunity. Early clinical translation is underway, including HLA‐A2–specific CAR‐Tregs for kidney and liver transplantation (TX200/STEADFAST and QEL‐001), and recent preclinical work highlights synergy between HLA‐A2‐CAR‐Tregs and costimulation blockade in allograft models [1, 2, 3, 4, 5]. Foundational preclinical and translational studies have also demonstrated antigen‐specific CAR‐Tregs across transplantation [6, 7, 8, 9, 10], autoimmunity [11, 12, 13, 14], and neuroinflammation [15, 16].

Current CARs, however, inherit design choices from cytotoxic T‐cell applications notably the use of single‐chain variable fragments (scFvs) cloned from existing monoclonal antibodies (mAbs). A growing body of evidence shows that scFv biophysical and structural features including affinity, hydrophobic patches and charge distribution, framework stability, aggregation propensity, and epitope geometry shape basal (“tonic”) signaling, phenotypic stability, and exhaustion [17, 18, 19, 20]. These relationships are especially salient for Tregs, in which excessive or qualitatively inappropriate signaling can erode suppressive identity; indeed, high‐affinity CAR engagement can skew human FOXP3^+^ Tregs toward a pro‐inflammatory program, whereas tempering proximal signaling or lowering CAR affinity improves Treg‐appropriate function [21, 22].

At the same time, the CAR backbone and expression level establish the cell's fundamental operating mode. Intracellular signaling domains, genomic context, and surface density can dominate tonic signaling and lineage stability, whereas the scFv tunes signal input within that architecture a “signal thermostat” rather than a passive targeting moiety. This hierarchy is particularly relevant in CAR‐Tregs, because host‐derived costimulation can in some settings partially compensate for limited CAR‐intrinsic costimulation, while backbone choice can either preserve or destabilize Treg identity [8, 9, 10, 23, 24, 25, 26, 27].

We therefore posit that phage‐display–selected scFvs, purpose‐screened for regulatory performance criteria, can be used to explore the CAR‐Treg design space more systematically. Modern phage display enables selection from vast, fully human or semi‐synthetic libraries and supports counter‐selection for developability and context‐relevant binding properties [28, 29, 30]. In practical terms, phage display allows rapid, scalable, library‐based generation of diverse scFv panels and iterative panning under defined selection and counter‐selection pressures. Importantly, we do not treat “low‐tonic” behavior as an intrinsic property of phage display. Rather, the central hypothesis is that a deliberately configured multi‐parameter selection campaign can enrich scFvs with biophysical features that, in defined CAR architectures, reduce clustering‐prone behavior and favor Treg stability.

This phage‐first concept must also be distinguished from adjacent optimization strategies. Similar goals can be pursued by retrospective engineering of legacy mAb‐derived scFvs, by controlling CAR density through promoter tuning or targeted integration, or by using alternative binding scaffolds such as nanobodies, peptide binders, DARPins, or receptor ectodomains. We acknowledge the validity of these routes. Our argument is narrower: phage‐first discovery uniquely enables prospective co‐optimization of specificity, developability, and hypothesized signaling behavior before CAR construction, potentially reducing the need for post hoc “repair” of pre‐existing binders. Conceptually, this strategy still builds on the original CAR “T‐body” principle of coupling antibody‐derived recognition to T‐cell signaling but redirects that design logic toward regulatory rather than cytotoxic cell states [31].

**FIGURE 1:**
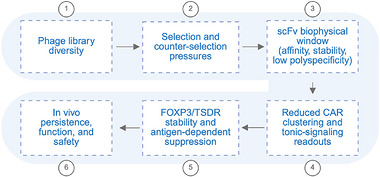
**Hypothesized causal chain linking phage‐first binder discovery to CAR‐Treg function**. Phage display is used here as an iterative in vitro selection platform in which large scFv libraries are enriched for target binding while counter‐selection can be applied against undesired properties such as polyspecificity, hydrophobicity, self‐association, or poor framework stability. The phage‐first concept is therefore not proposed to improve CAR‐Tregs by scFv origin alone; rather, its value lies in rapid, scalable, library‐based generation of diverse scFv panels and programmable selection/counter‐selection for biophysical properties hypothesized to influence CAR clustering, tonic signaling, and Treg lineage stability. The proposed causal chain links selection pressure to measurable scFv biophysical profiles, CAR clustering and antigen‐independent tonic signaling, FOXP3/Treg‐specific demethylated region (TSDR) stability, suppressive function, and ultimately in vivo persistence and safety. These links are presented as testable hypotheses, not as demonstrated properties of phage display itself. Data obtained from CAR‐Treg testing should feed back into subsequent library design, panning stringency, and counter‐selection criteria.

In this Ideas and Speculations article, we therefore recast the agenda as an illustrative two‐step, falsifiable framework. Figure 1 summarizes the proposed causal chain, and Table 1 distils the framework into representative questions, readouts, and decision criteria. Phase 1 focuses on biophysical triage and proof‐of‐concept; Phase 2 tests mechanism, design hierarchy, and translational performance in vivo. As an Ideas & Speculations contribution, this article does not present primary research data; instead, it formulates testable hypotheses and a practical framework for binder discovery and CAR design tailored to CAR‐Tregs.

**TABLE 1:** **Two‐phase framework for testing phage‐first CAR‐Treg binder discovery**. Phase 1 prioritizes biophysical triage and proof‐of‐concept testing of candidate scFvs before in vivo work. Phase 2 tests whether favorable binder‐associated properties remain interpretable across CAR backbone architectures, expression‐control strategies, and disease‐relevant models. The comparison between legacy mAb‐derived scFvs and phage‐display‐generated scFv panels should not be interpreted as treating scFv origin as a physical variable per se; rather, it tests whether different discovery workflows enrich for distinct biophysical property profiles that perform differently in matched CAR‐Treg contexts. The term “tonic window” denotes a range of low antigen‐independent CAR signaling compatible with preserved FOXP3 expression, TSDR demethylation, and antigen‐dependent suppressive function; it does not refer to physiological tonic TCR signaling required for Treg maintenance. Abbreviations: CAR, chimeric antigen receptor; scFv, single‐chain variable fragment; FOXP3, forkhead box P3; TSDR, Treg‐specific demethylated region; NFAT, nuclear factor of activated T cells; pCD3ζ, phosphorylated CD3ζ.

Phase	Priority question	Illustrative readouts	Decision criterion
**Phase 1**	Do candidate scFvs combine acceptable CAR‐Treg‐relevant developability with low tonic signaling and preserved Treg identity?	Biophysical triage (e.g., stability, self‐association, hydrophobicity, polyspecificity), antigen‐free tonic signaling readouts (e.g., NFAT/Nur77, pCD3ζ), FOXP3 protein, TSDR demethylation, antigen‐dependent suppression, orthogonal off‐target screens	Candidates should show a favorable balance of low tonic signaling, preserved lineage stability, retained antigen‐dependent suppression, and limited polyspecificity before further prioritization.
**Phase 1**	How does the optimal signaling window shift across affinity and antigen‐density contexts?	Affinity × density mapping, cytokine drift, repetitive stimulation, inflammatory challenge, FOXP3/TSDR stability	Candidates should remain functionally stable across relevant signaling contexts; findings should define a practical “tonic window” rather than a single universal optimum.
**Phase 2**	Does the proposed scFv‐associated advantage remain interpretable across different CAR architectures and expression‐control settings?	Discovery workflow and resulting scFv property profile × intracellular architecture × expression level; CAR density, clustering proxies, tonic signaling, FOXP3/TSDR stability, suppression	Further prioritization is justified only if the binder‐associated benefit remains evident within defined backbone and expression contexts.
**Phase 2**	Do prioritized constructs retain benefit in vivo and support translationally meaningful tolerance endpoints?	Persistence, tissue localization, efficacy, safety, immunosuppression taper or disease‐relevant functional endpoints	Progression is justified only if the proposed benefit translates in vivo without evidence of unintended systemic immunosuppression.

## 2 A Practical Two‐Phase Framework to Test the central Hypothesis

Most CAR binding domains come from monoclonal antibodies originally optimized for cytotoxic applications. We therefore separate established observations from testable hypotheses and organize the agenda as an illustrative two‐step, falsifiable framework. Specifically, we hypothesize that phage‐selected scFvs can help define a CAR‐Treg “tonic window” compatible with preserved FOXP3/TSDR stability and safe, antigen‐dependent regulation.

### 2.1 Phase 1 — Biophysical Triage and Proof‐of‐Concept

The first task is to identify candidate scFvs whose biophysical properties justify cell‐based testing. Phage display is an in vitro selection technology in which large libraries of antibody fragments are displayed on bacteriophage particles and enriched through iterative rounds of target binding, panning, and counter‐selection. In this context, developability must be defined pragmatically for a living CAR‐Treg product rather than for an injectable antibody. For early discovery, this means avoiding obvious liabilities that could compromise CAR expression, promote antigen‐independent signaling, or create unacceptable off‐target immunosuppression. Low polyspecificity is therefore particularly important, and obvious framework instability may increase the risk of clustering‐prone CAR behavior. These considerations make phage display attractive because target binding can, in principle, be combined with simple counter‐selection against nonspecific or unstable binders.

Phase 1 should therefore remain deliberately simple. Diverse human scFv panels generated by phage display should first be prioritized by target binding, sequence plausibility, absence of obvious polyreactivity signals where tested, and compatibility with CAR surface expression. Candidate binders should then be inserted into matched CAR constructs to test whether a rapid, library‐based phage‐first workflow yields scFv panels with biophysical and functional profiles that differ from legacy mAb‐derived binders under controlled backbone and expression conditions. Antigen‐free tonic readouts, for example Nur77/NFAT reporters or phosphorylated CD3ζ (pCD3ζ) can be interpreted independently of backbone changes [17, 18, 19, 20, 21]. At this stage, prioritization should require a favorable balance of low tonic signaling at matched CAR density, preservation of FOXP3 protein and Treg‐specific demethylated region (TSDR) status as an epigenetic marker of stable Treg lineage identity under inflammatory stress, retained antigen‐dependent suppression, and no obvious specificity concern. More extensive biophysical or off‐target profiling can be reserved for shortlisted candidates moving toward translational development. Failure on these criteria would argue against assigning high priority to a candidate for further mechanistic or in vivo evaluation. Because epigenetic lineage stability is central to durable regulation, TSDR should be treated not merely as a descriptive marker but as a mechanistic readout; targeted demethylation studies underscore that FOXP3/TSDR state can influence physiological FOXP3 expression and, in selected contexts, suppressive capacity [32, 33].

A second Phase‐1 task is to map the CAR‐Treg tonic window across affinity and antigen density [34, 35]. Here, “tonic window” denotes a range of low antigen‐independent CAR signaling compatible with preserved FOXP3 expression, TSDR stability, and antigen‐dependent suppressive function; it does not refer to physiological tonic TCR signaling required for Treg maintenance. This matrix is unlikely to yield a single universal optimum. In high‐density settings such as alloantigen recognition, lower‐affinity binders may improve specificity while limiting tonic risk; in autoimmune settings, where relevant antigens may be sparse or tissue restricted, somewhat higher affinity may be required, at the cost of a narrower safety margin. More broadly, clinical‐facing syntheses of engineered T‐cell design identify affinity and tonic‐signal tuning as major performance levers across CAR platforms [36]. Repetitive stimulation and inflammatory challenge should therefore be built into Phase 1 to determine whether candidate scFvs preserve FOXP3/TSDR stability across disease‐relevant signaling environments [5, 21, 26]. A related translational caveat is that outside well‐defined alloantigens such as HLA‐A2, discovery of sufficiently tissue‐restricted targets may itself become rate‐limiting for broader application.

### 2.2 Phase 2 — Mechanistic and Preclinical Validation

Phase 2 should test whether favorable scFv properties remain beneficial once the broader CAR design hierarchy is varied. The purpose of this second phase is not to broaden the thesis beyond binder discovery, but to determine under which architectural and expression contexts a binder‐level advantage can be meaningfully observed. The key experiment is factorial: compare discovery workflow and resulting scFv property profile across distinct intracellular architectures and expression‐control strategies, then invert the comparison by holding the architecture constant while varying the binder. Relevant variables include co‐stimulatory domain family, first‐generation versus signaling‐augmented designs where appropriate, targeted versus random integration, and promoter strength or other density‐control modules [1, 8, 9, 10, 24, 25, 26, 27, 37, 38, 39, 40]. Readouts should include CAR density, clustering proxies, tonic signaling, FOXP3/TSDR stability, suppression, and cytokine drift. This design makes it possible to ask whether the scFv genuinely lowers tonic signaling within a permissive backbone, or whether backbone‐driven operating modes dominate the phenotype.

Only after this mechanistic hierarchy has been defined should selected constructs move to in vivo evaluation. At this stage, the key question is not whether phage‐selected scFvs improve every aspect of CAR‐Treg biology, but whether any binder‐associated advantage remains detectable in relevant disease settings. Accordingly, head‐to‐head comparisons between mAb‐derived and phage‐selected scFvs should be performed in matched backbones and, where informative, alongside combination regimens already implicated in CAR‐Treg biology, such as costimulation blockade or engineered IL‐2 support cues [1, 7, 23, 40]. Relevant endpoints include persistence, tissue localization, functional efficacy, and safety across transplantation, autoimmunity, and tissue inflammation [1, 2, 3, 4, 5, 6, 7, 8, 9, 10, 11, 12, 13, 15, 23, 24, 25]. For transplantation, pragmatic translational metrics include CAR‐Treg engraftment, CAR‐Treg localization in target tissue biopsies, recipient hyporesponsiveness toward donor antigens, and tapering of conventional immunosuppression without rejection [3, 4, 5]. For broader inflammatory indications, equivalent disease‐relevant functional endpoints should be paired with explicit monitoring for unintended systemic immunosuppression. Together, these studies would determine whether a putative scFv‐level advantage remains interpretable beyond the bench and under biologically realistic conditions.

## 3 Conclusion

Our central claim is therefore not that phage display automatically produces superior CAR‐Treg binders, but that it provides a tractable way to test a more ambitious design logic. The broader architectural variables discussed here are therefore not intended to displace the binder‐centered hypothesis, but to define the contexts in which a phage‐selected scFv advantage can be unmasked, quantified, or falsified. By prospectively tuning affinity, epitope geometry, polyspecificity, and framework stability, phage‐first discovery may expand the repertoire of scFvs that are compatible with regulatory rather than cytotoxic cell states.

The immediate priority is not clinical extrapolation but disciplined comparison: phage‐selected versus mAb‐derived binders in matched backbones, controlled expression contexts, and explicitly defined signaling windows. If these experiments converge, phage‐selected scFvs could broaden the antigenic space accessible to CAR‐Tregs and make tolerance‐oriented cell therapies more predictable and scalable. In that sense, the value of phage display lies less in access to novel binders per se than in design freedom the ability to move beyond retrofitting cytotoxic tools and toward truly regulatory‐optimized CARs with measurable translational endpoints.

## Conflicts of Interest

F.N. and M.H.‐W. are inventors on intellectual property related to CAR technologies, including autoimmune‐relevant targets [25, 41]. The conceptual framework proposed here is broader than any single proprietary target or binder, but these relationships may intersect with parts of the translational landscape discussed and are therefore disclosed for transparency.

## Data Availability

Data sharing not applicable to this article as no datasets were generated or analysed during the current study.
